# CLINICAL OUTCOME AFTER INFECTED TOTAL KNEE AND TOTAL HIP ARTHROPLASTY

**DOI:** 10.1590/1413-785220162401150767

**Published:** 2016

**Authors:** Falk Mittag, Carmen Ina Leichtle, Michael Schlumberger, Ulf Gunther Leichtle, Markus Wünschel

**Affiliations:** 1. University Hospital Tübingen, Hoppe-Seyler-Str. 3, Tübingen, Alemanha.

**Keywords:** Infection, Hip, Knee, Arthroplasty, Bacteria.

## Abstract

**Objective:**

Infection after total hip (THA) and knee arthroplasty (TKA) is a serious complication which typically leads to a long lasting and intensive surgical and medicamentous treatment. The aim of this study was to identify factors that influence outcome after revision surgery caused by prosthetic infection.

**Methods:**

We retrospectively analyzed 64 patients who had revision surgery between 1989 and 2009 due to periprosthetic infection. We examined a total of 69 joints (TKA: 36%, THA: 64%), follow-up 5.1 years (0.5-21 years) after the initial surgical intervention. The mean patient age at time of surgery was 67 years old (43-79 years old). Clinical data and scores including the Western Ontario and McMaster Universities (WOMAC)-Index, the Harris Hip Score (HHS) and the Hospital for Special Surgery Score (HSS) were surveyed.

**Results:**

There was no difference in clinical scores regarding treatment between a single and a multiple stage treatment regime. Infections with multiple microorganisms and Enterococcus spp. lead to a significantly higher number of interventions. Using a modified Tsukayama system we classified 24% as type I, 34% type II and 42% type III- infections, with no differences in clinical outcome. Overweight patients had a significantly lower HHS and WOMAC-score. Immunosuppression leads to a worse WOMAC and HSS-Score. An increased number of procedures was associated to a limping gait.

**Conclusion:**

Thorough surgical technique leads to good clinical results independent of infection-type and treatment philosophy. ***Level of Evidence III, Case Control Study.***

## INTRODUCTION

Although infection rates after primary total knee arthroplasty (TKA) and total hip arthroplatsy (THA) are low ^1^, the numbers of revision arthroplasty caused by prosthetic infection is rising ^2^due to a growing quantity of arthroplasties performed. ^3^Revision arthroplasty because of prosthetic infection usually takes longer, often requires temporary removal of the implant and is associated with long-lasting administration of antibiotics thus being a serious cost factor in the health care system. In rare cases the infection can only be ceased by definite removal of the implant or an arthrodesis. Even if the infection can be controlled, clinical outcome is worse compared to revision arthroplasty in an aseptic situation regarding pain, function and activities of daily living. ^4^ ^,^ ^5^

Many factors have been identified that make patients more susceptible to an infection. Diseases like Diabetes mellitus, obesity, renal failure, rheumatoid arthritis, neoplasms and haemophilia ^6^ ^,^ ^7^as well as certain medications like cortisone or other immunosuppressive medication lead to higher infection rates.

The first step to initiate the right treatment is the correct diagnosis, which in this special entity is sometimes very difficult to achieve since there is no single evidentiary symptom, diagnostic test or imaging modality. In fact, only the combination of the latter in conjunction with clinical experience makes the diagnosis of a periprosthetic infection probable. Laboratory tests like C-reactive protein (CRP) or erythrocyte sedimentation rate (ESR) have good sensitivity but lower specifity and are mainly useful when a number of tests at different dates can be compared and correlated to clinical symptoms. Native x-rays in combination with other image modalities like radionuclide scans are also useful for the diagnosing process since magnetic resonance tomography and computed tomography may hardly be used due to artifacts caused by the implant. Because ultrasound can only detect effusion in the joint that might be caused by several other reasons, more invasive diagnostic methods should be used, namely the aspiration of the joint. ^8^If an aspiration is performed the causative agent can be isolated ideally, leading to a specific antibiotic therapy even before surgical treatment. The leukocyte count in the sample is another piece in completing the diagnostic process. Even though a preoperative aspiration might be sterile, intraoperative swabs and tissue samples, especially of the periprosthetic membrane, need to be taken. Histopathological examination and Polymerase chain reaction screening tests for bacterial desoxyribonucleinacid (DNA) have also been established to verify a prosthetic infection with the clear disadvantage that the results are available only postoperatively. When it comes to treatment there are several options. Not rarely the prosthesis has to be temporarily removed to control the infection whereas in other cases the arthroplasty can be maintained due to thorough debridement and irrigation, partly exchanging the components and a long lasting antibiotic therapy. Exclusive or concomitant antibiotic therapy and usage of vacuum-systems, continuous drainage, antibiotic beads-and individualized bone cement are concomitant measures. 

The decision whether to keep or remove the prosthesis is usually based on the type of infection utilizing the Tsukayama classification. ^9^While in short lasting infections, especially after primary arthroplasty, chances are good to preserve the implant. In the case of a chronic infection the bacteria has colonized the implant which usually makes it necessary to remove it in order to control the infection. The acute haematogenous type of infection may happen years after primary arthroplasty. In these cases an attempt to maintain the implant might be done if the implant and the bone stock are intact and clinical symptoms are acute. While in primary arthroplasty many aspects have been identified that have impact on the clinical results, the aim of this study was to identify patient- and treatment related factors that influence the outcome after revision arthroplasty caused by prosthetic infection and to give a comprehensive overview of the topic.

## MATERIALS AND METHODS

One hundred and sixty seven patients (78 females, 89 males) with 79 total knee and 104 total hip arthroplasties were surgically treated because of an infected implant between 1989 and 2009. All patients were contacted and 64 (41 males, 23 females) patients with 69 infected joints (TKA: 36%, THA: 64%) could be included in the study. 

The hospital charts were analyzed and the relevant data was extracted. ( Tables 1and ^2^) Clinical scores including the Western Ontario und McMaster Universities (WOMAC)-Index in the German version ^10^ ^,^ ^11^, the Harris Hip Score (HHS) ^12^and the Hospital for Special Surgery Score (HSS) ^13^were used to rate clinical outcome. A modified Tsukayama-classification defining a type-I infection to occur within 3 months after primary arthroplasty was used to classify the infections. A body mass index (BMI) below 18 kg/m², 18 kg/m² to 25 kg/m² and above 25kg/m² was defined as under-, normal- and overweight, respectively. Statistical analysis was performed with SAS jmp^(r)^ - Statistical Discovery Software utilizing the Wilcoxon-Mann-Whitney test and the Spearman's rank correlation coefficient were applicable.

**Table 1:** Patient related parameters.

Gender
Age at first revision surgery
Body mass index
Other diagnosis
Regular medication
Time elapses from primary arthroplasty to revision arthroplasty
Tsukayama classification
Isolated microbiologic pathogens
C-reactive protein, erythrocyte sedimentation rate
White blood cell count in pre-operative joint puncture

**Table 2:** Treatment related parameters.

Duration of antibiotic therapy
Length of hospital stay
Número de procedimentos realizados até o controle da infecção
Intraoperative details (vacuum, debridement, suction-drainage, antibiotic beads) etc
Arthroplasty models used for revision arthroplasty
Duration of time-period between removal of implant and revision arthroplasty

The research presented in this work conforms to the Helsinki Declaration and to local legislation. It has been approved by the Ethical Committee (31/2010BO2). Informed consent was obtained from all participants in accordance with the Helsinki Declaration.

## RESULTS

Results for THA and TKA are presented together. In case of relevant differences between THA and TKA results are shown separately. The mean patient age at time of surgery was 67 years (43-79). We examined a total of 69 joints (TKA: 36%, THA: 64%) 5.1 years (0.5-21) after the first surgical intervention which consisted either of single- (23%) or multiple-stage treatment (77%). When only one procedure was performed the implant was never exchanged whereas multiple procedures always were associated with a temporary removal of the implant.

**Figure 1:**
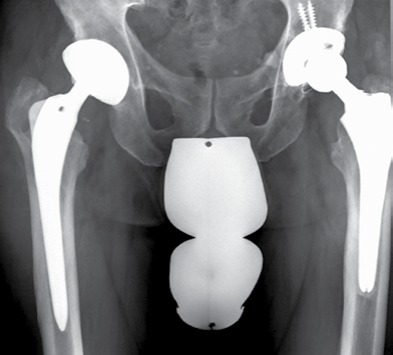
Preoperative x-ray of a 65-year old male patient 5 and 12 years after primary arthroplasty on the left, respectively right side. There are osteolytic bony changes in the region of the greater trochanter.

A typical patient case is illustrated by Figures 1- ^5^. Infection control was achieved in 100%. All but 5 patients had revision arthroplasty (4 THA-patients had a permanent girdlestone-situation and 1 TKA-patient was treated with an arthrodesis of the knee joint, this was due to patient preference). 37% of the patients suffered from a potentially immunosuppressive disease like diabetes *mellitus*, rheumatoid arthritis or a neoplasm. The course of the CRP and range of motion (ROM) for THA and TKA is illustrated by Figure 6. CRP was elevated preoperatively in 95% of the cases. Using the modified Tsukayama system we classified 24% as type I, 34% as type II and 42% as type III- infections with no difference in clinical outcome. In type I the implant could be maintained in 60% of the cases while in type II and III the rate was 23% and 7%, respectively with no differences between THA and TKA. The average number of procedures performed on each patient was 3 (1-15), average hospital stay was 42 days (10-190) and the average time period between arthroplasty and infection-caused revision surgery was 2.6 years (2 weeks - 12 years) with no significant differences between THA and TKA. The minimum preoperative leukocyte count in the aspiration fluid of the joint was 13690 /µl (average: 61948 ± 53799/µl, n=21). If the Leukocyte count in the joint fluid was elevated (>10000/µl) a microorganism could be cultured in 95% of the cases.

**Figure 2:**
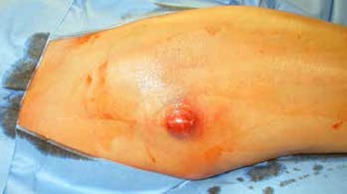
Abscess formation at the distal part of the scar shortly before perforation through the skin.

**Figure 3:**
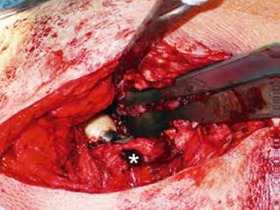
Intraoperative photo of the situs. The asterix denotes the large osteolyses in the greater trochanter.

**Figure 4:**
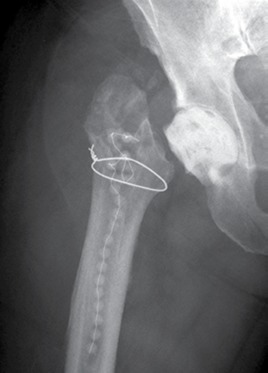
Postoperative x-ray reveals the antibiotic beads and the cement spacer after removal of the infected implant.

**Figure 5:**
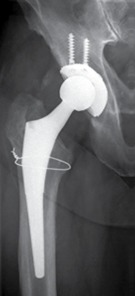
X-ray three years after revision arthroplasty shows well osteointegrated cup and stem. The osteolyses of the trochanter have filled up with bone.

**Figure 6:**
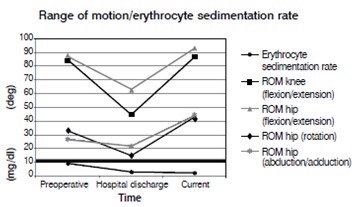
ROM and CRP at the indicated dates. CRP drops to normal patient levels, ROM improves after revision surgery.

**Figura 7:**
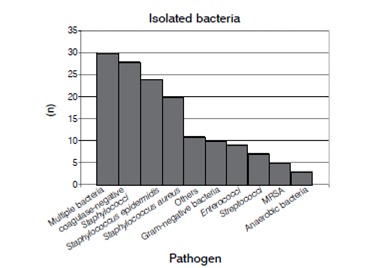
Cultured bacteria via joint aspiration or intraoperative swab.

*Staphylococcus spp.*were most frequently isolated (70%), followed by gram-negative microorganisms (14%) and *Enterococcus spp.*(13%). An overview of the isolated bacteria is given in Figure 7.

Infections with multiple microorganisms (p=0,0212, apparent in 43%) or *Enterococcus spp. *(p=0,0004) lead to a significantly higher number of interventions and thus to a longer hospital stay. Average duration of antibiotic therapy was 89 days (36 to 330, median:70). Duration of antibiotic treatment was also strongly dependent on the type of the isolated bacteria with significantly longer duration for *Staphylococcus epidermidis*(p=0,0429), *Enterococci*(p=0,0060) and gram-negative bacteria (p=0,0465) ( Table 3). In 57% of the surgeries of the hip a vacuum-sealing or a suction drainage was installed, in 41% antibiotics beads were administered. In all surgeries a thorough debridement including high-pressure lavage was used and the removable components (head, inlay) were exchanged during all single-stage procedures. The average time elapsed before revision arthroplasty was performed was 3.8 months (1.6 to 19). This interval was significantly longer (4.4 *vs*. 3.4 months) if more than one microorganism was involved. Hip joint revision arthroplasty was performed using a revision stem and -cup in 75% respectively 69%, for revision total knee arthroplasty a ligament sacrificing model was implanted in 65% of the cases. A higher number of surgeries of the hip was associated with Trendelenburg-limping. While limping patients had an average of 3.9 procedures, non-limping patients were operated only 1.4 times (p=0.009).

**Table 3:** Median and mean duration of antibiotic therapy until infection control was reached (no significant differences between THA and TKA).

Species	Median (days)	Mean (days)
*Staphylococcus epidermidis*	80	111 (± 85)
Gram-negative pathogens	93	139 (± 99)
*Enterococci*	150	187 (± 117)
Average for all	70	89 (± 64)

Average scores for WOMAC, HHS and HSS were 58±27, 70±20 and 69±13, respectively. The three types of infection did not significantly differ regarding the scores nor had the type of treatment a significant influence (retaining the implant vs. multiple stage revision surgery). 79% of our patients were overweight, which lead to a significantly lower HHS (p=0,0018) and WOMAC Score (p=0,0203) The patients being under immunosuppressive medication had significantly worse outcome regarding the WOMAC (p=0,0117) and the HSS-score (p=0,0056). 

## DISCUSSION

Septic complications after THA and TKA are rare but have severe implications on the patient. Long lasting hospitalization, continuous antibiotic therapy and multiple surgeries are usually part of the standard therapeutic regime. ^8^ ^,^ ^14^If the implant has to be removed temporarily the patient often loses the ability to ambulate autonomously and may need to stay at a nursing home until the revision arthroplasty can be performed. Calculations of the cost for revision arthroplasty have shown the immense amounts of money that is needed to treat a patient with an infected artificial joint. ^15^In the environment of scarce resources in the health care system combined with already high and growing number of primary arthroplasty performed it is necessary to analyze the typical course of periprosthetic infections, identify patients who are susceptible to unfavorable outcomes and find treatment options to optimize the results. 

Our data show the HSS, WOMAC-index and HHS did not differ between the three types of infections nor between the way they were treated and are comparable to other published data. ^16^In their study Tsukayama et al. included only hip joints and had a similar HHS for type I and III infections whilst others found different outcomes. ^9^Our data indicate that various types of infections were associated with a different rate of maintained joints during treatment. While in type-I infections 60% of the implants could be left in situ, this rate dropped to 23% and 7% for type II and III, respectively with no differences between THA and TKA. Based on this the surgeon should make a try to maintain the implant in acute and selected chronic infections taking into account the shorter hospital stay, less limping and equally high clinical scores. Even if this fails, the implant can be removed and switched to a multiple stage regime. Although in the literature the best results to control the infection are published for two stage revision arthroplasty with multiple debridements ^17^ ^,^ ^18^and this is certainly the safest way, the above mentioned side effects need to be taken into account. If either by aspiration or arthroscopic bioscopy the causing microorganisms are known before revision surgery some surgeons propose a single stage procedure with specifically produced antibiotic bone cement and immediate revision arthroplasty. If multiple microorganisms or *Enterococcus spp. *are identified, the implant should be removed due to the expected long lasting treatment regime and the high probability for removal in the course of treatment. This should especially be taken into account for the expected growing number of vancomycin resistant *Enterococci.*The number of microorganisms cultured strongly correlated with the amount of time passed before revision arthroplasty could be performed. The average time in our study was 3.8 months (1.6 to 19), which is in concordance with the literature. Most authors propagate revision surgery at least 8-10 weeks after implant removal (6 weeks of antibiotic treatment, 2 weeks cessation of therapy, then joint aspiration). ^14^The reason for the wide time-range for revision arthroplasty might be due to patient preference, organizing structures regarding admission and the fact that many patients were in a critical medical condition with need to optimize their status before surgery. During the course of treatment the CRP was highly elevated preoperatively and could be reduced by infection control to preoperative values as has been published by others ^19^emphasizing its importance in the context of periprosthetic infections. Our data also indicate that an elevated leukocyte count of > 10000/µl in the joint fluid is a predictor for a joint infection since in 95% of the cases bacteria could be cultured. In the 5% of cases with no cultured bacteria our cut off for infection would be an elevated leukocyte count of >25000 /µl in the joint fluid and/or other clinical data suggesting infection like elevated CRP/blood leucocytes or local abnormalities.

The progression of the ROM for TKA and THA is in concordance with other publications and shows even slightly higher values after revision arthroplasty compared to preoperatively which might be explained by the pain due to the infection.

Since performing surgery in obese patients is technically demanding due to anatomic conditions it has been shown that obesity is associated with a higher rate of complications after revision- and infections after primary arthroplasty. We found a significantly lower HHS and WOMAC-score in overweight patients conforming to the WHO-definition (BMI>25 kg/m²). Patients under immunosuppressive medication had significantly lower WOMAC and HSS-scores. This is in concordance with several other studies that have identified this subgroup performs lower due to their underlying disease and the immunocompromised status. ^20^

## CONCLUSION

Thorough surgical technique leads to good clinical results independent of infection-type and treatment philosophy. In Type-I and -II infections we recommend to try to maintain the implant during the first surgical intervention. Obesity, immunosupression, multiple pathogens and a high number of surgical procedures were identified as risk factors for a disadvantageous clinical outcome.
